# A Sub-Clustering Algorithm Based on Spatial Data Correlation for Energy Conservation in Wireless Sensor Networks

**DOI:** 10.3390/s141121858

**Published:** 2014-11-18

**Authors:** Ming-Hui Tsai, Yueh-Min Huang

**Affiliations:** Department of Engineering Science, National Cheng-Kung University, No.1, University Road, Tainan City 701, Taiwan; E-Mail: coluxtsai@gmail.com

**Keywords:** WSNs, energy conservation, data correlation, prediction model, virtual cluster

## Abstract

Wireless sensor networks (WSNs) have emerged as a promising solution for various applications due to their low cost and easy deployment. Typically, their limited power capability, *i.e.*, battery powered, make WSNs encounter the challenge of extension of network lifetime. Many hierarchical protocols show better ability of energy efficiency in the literature. Besides, data reduction based on the correlation of sensed readings can efficiently reduce the amount of required transmissions. Therefore, we use a sub-clustering procedure based on spatial data correlation to further separate the hierarchical (clustered) architecture of a WSN. The proposed algorithm (2TC-cor) is composed of two procedures: the prediction model construction procedure and the sub-clustering procedure. The energy conservation benefits by the reduced transmissions, which are dependent on the prediction model. Also, the energy can be further conserved because of the representative mechanism of sub-clustering. As presented by simulation results, it shows that 2TC-cor can effectively conserve energy and monitor accurately the environment within an acceptable level.

## 1. Introduction

As the rapid advances recently in wireless communications and sensor technologies, wireless sensor networks (WSNs) have emerged as the promising solution for various applications due to their low cost and easy deployment. A wireless sensor network consists of sensor nodes spatially deployed over a geographical area for monitoring physical environment. These sensor nodes are mainly composed of four components: power unit for power supply, sensing unit for data acquisition (sampling), processing unit for data processing, and radio unit for wireless communication. Typically, their limited power capability, *i.e.*, battery powered, make WSNs encounter the challenge of extension of network lifetime. Many interesting literatures [1–5] have proposed how to address this kind of problem. Therefore, it is a key issue in the design of systems based on WSNs that how to reduce the energy consumption and further prolong the network lifetime.

With regard to the energy conservation, Anastasi *et al.* [4] identify three main enabling techniques, namely, duty cycling, data driven, and mobility-based approaches. The first category exploits the power management (including mechanisms on MAC layer, network layer and cross layers) for energy saving by considering duty cycling [6–9]. To this end, various criterions can be used to decide which/when sensor nodes should be active. It is a well-known fact that the radio unit is the dominant part of energy consumption in a sensor node [10]. Hence, it has been shown in previous works [10,11] that an idle sensor node should turn its radio off to avoid unneeded energy waste, namely avoid idle listening. In [12], the authors adapt the scheduled rendezvous scheme for duty cycling. The basic idea is that each node should wake up based on a wakeup schedule. They utilize the data gathering tree structure to achieve both energy efficiency and low packet delivery latency. The scheme staggers the active/sleep schedule of the nodes in the data gathering tree according to its depth in the tree. The second category focuses on sensor readings and can be further be subdivided into two main classifications: data reduction and energy-efficient data acquisition. The former adopts in-network processing, such as aggregation [13,14], compression [15] or prediction [16,17], in order to reduce the amount of data readings that need to be transmitted. Clearly, it can perform energy conservation that is benefitting from cutting down the transmissions. However, it has to pay more attention to keep the sensing accuracy within an acceptable level for the application, since data reduction is designed to reduce the amount of communication. The other proposes adaptive sampling schemes [18] for energy saving while especially taking energy hungry devices into consideration. Furthermore, unneeded and reducible sampling results in useless energy consumption because they bring about unneeded communications. The last category considers the specific schemes that the mobility of sensors nodes or sink is available. By introducing mobility in WSNs, routing schemes for the collection of data reading should take the mobile pattern of sensors nodes into consideration.

As mentioned above, a lot of studies on the WSNs have been dedicated to energy conservation. WSNs using hierarchical protocols are partitioned into clusters with different concerns [19]. Many hierarchical protocols show their better ability of energy efficiency, stability, and scalability [20,21] in WSNs. The cluster head can not only be one of the coordinators among the inter-cluster communication, but also be a key role for in-network processing within the intra-cluster. Besides, data reduction based on the correlation of sensed readings can efficiently reduce the amount of required transmissions. Therefore, we use a sub-clustering procedure based on spatial data correlation to further separate the hierarchical (clustered) architecture of a WSN. The representative mechanism (data reduction) is also engaged in these sub-clusters for more energy conservation. In our algorithm, all sensor nodes are first grouped into clusters (physical clusters) using hierarchical protocols. Subsequently, a sensor node generates a prediction model to forecast the data readings instead of reporting periodically data readings. The prediction mechanism is based on temporal data correlation demonstrated in [16]. The forecasting value is regarded as the concrete data reading. Hence, the transmissions of data reading reports are rationally reduced. Afterwards, sensor nodes within a physical cluster having strong spatial data correlations are then grouped into a sub-cluster (or say virtual cluster). Those chosen sensor nodes become the representative nodes for the sub-clusters and bear the responsibility of sensing environment around the whole virtual cluster.

**Figure 1.**
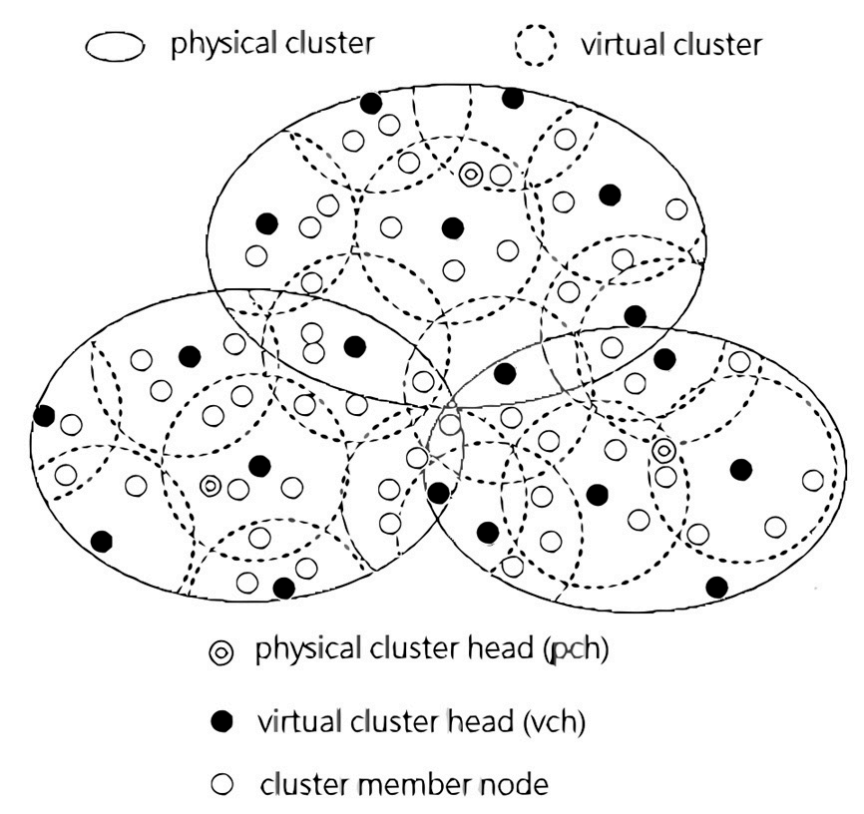
Two tiers hierarchical architecture is composed of physical and virtual clusters.

The main contributions of this paper are twofold. First, we use two tiers hierarchical architecture to form physical clusters and virtual clusters as shown in Figure 1. Under this kind of architecture, the energy conservation benefits by the reduced transmissions, which are dependent on the prediction model. Also, the energy can be further conserved because of the representative mechanism by sub-clustering. Only the representative sensor node of the virtual cluster turns its sensing unit on. Oppositely, the others within a virtual cluster can just power off for energy saving. Secondly, the decrement of transmitted data readings results in the possible decreased accuracy in monitoring environment. Moreover, the representative sensor nodes need to be able to represent the monitoring area well. Therefore, we try to keep the accuracy of monitoring within an acceptable level. Note that the representative sensor needs to transmit to physical cluster head (abbreviated as *pch*) its readings, which differ from the predicted readings by more than a certain pre-specified error threshold.

The remainder of this paper is organized as follows. Section 2 reviews several hierarchical protocols. Also, some data reduction algorithms, based on correlation and prediction proposed previously, are surveyed in a concise manner. In Section 3, we put forward the system model of our scheme. A sub-clustering algorithm under a hierarchical architecture is presented. Also, we investigate our data reduction mechanism based on data prediction and correlation. Then, the simulation results of the proposed algorithms are presented in Section 4. Finally, conclusion is given in the last section of the paper.

## 2. Related Work

A survey of energy conservation in WSNs is given in [4]. In order to efficiently reduce the energy consumption and further prolong the network lifetime, we focus on the literatures of energy conservation, especially those hierarchical protocols with concern on energy efficiency and data reduction schemes using data correlation and prediction.

Certainly, the hierarchical architecture is introduced to WSNs because it has proven to be an effective approach to provide better energy efficiency. As network nodes are organized in clusters, the router nodes are responsible for coordinating activities within the cluster and forwarding data readings between clusters. The use of routing hierarchy has many advantages, including better energy efficiency and scalability. LEACH (Low Energy Adaptive Clustering Hierarchy) [22] is the most famous algorithm that uses clustering to prolong the network lifetime. From the energy perspective, it uses randomized rotation of the cluster heads to distribute the energy consumption among the sensor nodes in a network. Many research efforts in energy efficiency follow LEACH with different concerns, such as cluster head selection, clustering cost, and cluster maintenance. In [23], the authors propose an improved routing algorithm of ring based multi-hop clustering (IRBMC) to prolong the lifetime of network. WSNs are divided into heterogeneous spacing rings to control the cluster number and build unequal size of clusters in different rings. The cluster heads selection is depending on the residual average energy estimation, which is taken into account to balance energy consumption of nodes in each ring. Also, in [24], two integer linear program (ILP) formulations are proposed for assigning sensor nodes to clusters in a two-tiered network where the relay nodes are used as cluster heads. The objective of ILP formulations is to load balanced clustering, that is, to maximize the lifetime of the relay node network by distributing the total load for data communication over the different relay nodes.

Data reduction techniques are designed to reduce the amount of transmissions, including data aggregation [25,26], compression, and prediction [16,17]. In [26], the authors propose a data correlation-based clustering (DCC). In each round of data acquisition, data readings of all nodes in one cluster are gathered at the cluster head, on which data readings will be compressed before send to the sink. The sink mines data correlations from historical data to construct clusters. However, the network is divided into several disjoint clusters by minimum degree first clustering, which is irrelevant with data correlation. That is, the data correlation between the cluster members can't be substantiated. In [16], PREMON paradigm trades increased computation (for deriving prediction models) for savings in number transmissions. In [17], the authors propose a buddy protocol that sensor nodes with high temporal correlation formed a buddy group. The nodes within the same buddy group (cluster) take turns keeping their radio on. The representative node responds on behalf of its buddies. However, GAF [27] is used for clustering in this protocol and the data readings between buddies have not been verified as being correlated. Therefore, the difference of data readings between the representative and buddies is not negligible.

In [25], the authors propose a distributed clustering-based aggregation algorithm for spatially correlated WSNs. When the clusters are constructed by the α-local spatial clustering, the data readings of cluster head and members have very high correlation. Only the cluster heads need to do the data sampling work. The data readings are then aggregated to a cluster head backbone and transmitted to the sink. Due to the lack of re-clustering mechanism, the constructed clusters based on weighted dominant set cannot accommodate themselves to the changing environment. Further, the cluster head has to keep sensing in order to monitor environment. Therefore, the cluster head tends to energy depletion.

## 3. A Sub-Clustering Algorithm Based on Spatial Data Correlation

Our proposed algorithm (two tiers clustering based on data correlation) abbreviated as 2TC-cor is composed of two procedures: the prediction model construction procedure and the sub-clustering procedure. Of course, the sensor network is partitioned into physical clusters by the distributed hierarchical routing protocols before the proposed procedures. We can choose LEACH, DCC, IRBMC, or others as the underlying hierarchical routing protocol due to the independence from those procedures. However, cluster heads, gateway nodes, backbone nodes, or the connected dominating set (CDS) play the key roles for the preservation of networks reachability in the hierarchical routing protocols. These nodes, called as router nodes, generally have to always switch their power on according to the adapted routing protocols. Therefore, it is important that the energy saving (power on/off) at router nodes is dependent on the underlying routing protocol rather than resulted from our sub-clustering procedure.

In this section, we will first introduce the construction of prediction models that are used to predict the data readings of sensor nodes. Each node within a physic cluster then attempts to establish buddy relationship with its neighbors during the sub-clustering procedure. The sensor nodes with high data correlation (or say buddies) are then divided into a virtual cluster and the chosen representative nodes bear the monitoring responsibility.

### 3.1. Constructing Prediction Models

Perhaps the readings of sensor nodes show high spatial and/or temporal correlation in the monitoring environment. We can predict a sensor node's reading based on the readings of the sensor nodes around it (spatial correlation) or based on itself historical readings (temporal correlation) [16]. Therefore, at the beginning of this procedure, each sensor node in a physical cluster calculates its prediction model according to its historical readings. The prediction models specify the readings at a sensor node as a function of its readings in the past. We suppose that a sensor node has enough memory to store the historical readings. Accordingly, sensor nodes generate their predictions by linear regression [28].

A sensor node *s* measures it's data reading *R_s_*(*t*) at time *t*. The prediction model of node *s* is denoted as *R̂_s_*(*t*). Over time, it collects a set of data readings (*R_s_*(*t*_1_), *R_s_*(*t*_2_),…, *R_s_*(*t_k_*)). With consideration of the characteristics of the application, we may pre-specify a set of basis functions, *F* = {*f*_1_(*t*), *f*_2_(*t*),…, *f_n_*(*t*)}. In regression, we would like to fit the basis functions *F* to those *k* data readings, that is, to find basis functions coefficients, *W* = (*w*_1_,*w*_2_,…,*w_n_*)^⊤^. Such that:

(1) $${\hat{R}}_{s}\left(t\right)=w_{1}f_{1}\left(t\right)+w_{2}f_{2}\left(t\right)+\cdots +w_{n}f_{n}\left(t\right)=\sum_{i=1}^{n}w_{i}f_{i}\left(t\right)\approx R_{s}(t)$$

where, *t* = *t*_1_,*t*_1_+t^*^,*t*_1_+2t^*^,…,t_k_, t^*^ is the frequency of reports requested based on the application and *t_k_* = *t*_1_+ (*k*−1)t^*^. To formulize the approximation, we use root mean squared error (RMSE) as the error metric. Hence, the parameters W^*^ are calculated in order to minimize the RMSE:

(2) $$W^{*}=\arg\;\underset{w}{\min}\sqrt{\frac{1}{k}{\sum_{i=1}^{k}\left(R_{s}\left(t\right)-{\hat{R}}_{s}\left(t\right)\right)}^{2}}=\arg\;\underset{w}{\min}\sqrt{\frac{1}{k}\sum_{i=1}^{k}(R_{s}\left(t\right)-\sum_{i=1}^{n}w_{i}f_{i}{\left(t)\right)}^{2}}$$

As presented in [28], the basis function coefficients *W* can be obtained.

**Figure 2.**
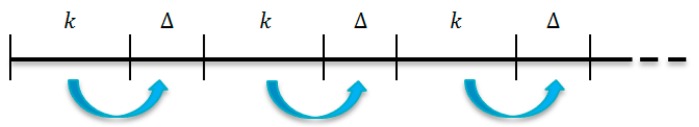
Using *k* concrete data readings to forecast the next Δ data readings.

Subsequently, we use the generated model *R̂_s_*(*t*) as the predictor for the next Δ readings where *t* = *t_k_*_+1_, *t_k_*_+2_, …, *t_k_*_+Δ_. That is to say, during the period (from *t*_1_+kt^*^ to *t*_1_+(*k*+Δ−1)t^*^), the forecasting value *R̂_s_*(*t*) is conditionally regarded as the concrete reading *R_s_*(*t*) of sensor node at the t^*^ interval. If the difference between *R̂_s_*(*t*) and *R_s_*(*t*) is within a certain pre-specified error threshold *ε*, the sensor node doesn't need to transmit its concrete reading to the cluster head (*pch*). The predicted value *R̂_s_*(*t*) is treated as the concrete reading of sensor node *s*. It means that the sensor node can reduce transmissions by using prediction model. On the contrary, if the difference is more than *ε*, the sensor node needs to send out its concrete reading to *pch*. Such a predicted value is termed as “violation” and the sensor node needs to transmit its concrete reading to *pch*. After Δ readings, the coefficients *W* are calculated once again using the last *k* readings, as illustrated in Figure 2. And the new obtained *W* is sent to *pch* and combined with *F* to forecast the next Δ readings.

### 3.2. Sub-Clustering to be Virtual Clusters

In order for more energy savings to keep fewer sensor nodes radio on, or say to avoid idle listening, we further partition (sub-clustering) the physical cluster based on the spatial data correlation of sensor nodes. The sensor nodes with high spatial data correlation are formed into a virtual cluster and only the representative nodes keep their radio on. Here, our sub-clustering algorithm is composed of two procedures: virtual cluster head selection procedure (VCHS procedure) and virtual cluster construction procedure (VCC procedure). Even though large number of sensor nodes may turn their radio off, the network reachability in our algorithm is still preserved since the underlying hierarchical routing protocol is responsible for it.

At the specified sample time stamp *t_k_, pch* has received the sets of concrete data readings (*R_s_*(*t*_1_), *R_s_*(*t*_2_), …, *R_s_*(*t_k_*)) of sensor node *s* ∈ *S* where *S* is the set of cluster members of *pch*. The communication hop counts between sensor nodes *s* ∈ *S* are one or more which is depending on the underlying hierarchical routing protocol. Since we intend to separate the set *S* into several virtual clusters depending on the spatial data correlation, the calculation of correlation tries to identify in what degree a node *x* ∈ *S* is correlated with its neighbors *y* = {*y*|*y* ∈ *N*(*x*)} where *N*(*x*) is denoted as the set of node *x*'s neighbors belonging to *S*. The data readings of *x* and y are denoted as (*R_x_*(*t*_1_), *R_x_*(*t*_2_), …, *R_x_*(*t_k_*)) and (*R_y_*(*t*_1_), *R_y_*(*t*_2_), …, *R_y_*(*t_k_*)), respectively. We define *d_xy_* as the difference (distance) between the data readings of node *x* and y by the Euclidean distance:

(3) $$d_{xy}=\sqrt{{\left|R_{x}\left(t_{1}\right)-R_{y}\left(t_{1}\right)\right|}^{2}+{\left|R_{x}\left(t_{2}\right)-R_{y}\left(t_{2}\right)\right|}^{2}+\cdots +{\left|R_{x}\left(t_{k}\right)-R_{y}\left(t_{k}\right)\right|}^{2}}$$

Then, the expected value of *d_xy_* is

(4) $$E\left(d_{xy}\right)=\bar{d_{x}}=\frac{1}{\left|N(x)\right|}\sum_{y\in N(x)}d_{xy}$$

where |*N*(*x*)| is the number of nodes in *N*(*x*). Also, the deviation of *d_xy_* is *D*(*d_xy_*):

(5) $$\begin{array}{c} D\left(d_{xy}\right)=E\left\{{\left[d_{xy}-E\left(d_{xy}\right)\right]}^{2}\right\}=\frac{1}{\left|N\left(x\right)\right|}\underset{y\in N\left(x\right)}{\text{∑}}{\left(d_{xy}-\bar{d_{x}}\right)}^{2}=E\left(d_{xy}^{2}\right)-{\left[E\left(d_{xy}\right)\right]}^{2}\\ =\frac{1}{\left|N\left(x\right)\right|}\underset{y\in N(x)}{\text{∑}}d_{xy}^{2}-{\bar{d_{x}}}^{2} \end{array}$$

In order to find out a measurement of spatial correlated weight for each node *x* ∈ *S*, we further define the weight *scw_x_* of node *x* as

(6) $$scw_{x}=\frac{{\left[\sum_{y\in N(x)}\left|d_{xy}-\bar{d_{x}}\right|\right]}^{2}}{{\left|N\left(x\right)\right|}^{2}D\left(d_{xy}\right)}=\frac{{\left[\sum_{y\in N(x)}\left|d_{xy}-\bar{d_{x}}\right|\right]}^{2}}{\left|N\left(x\right)\right|\sum_{y\in N(x)}{\left(d_{xy}-\bar{d_{x}}\right)}^{2}}$$

According to Cauchy-Schwarz inequality, we have

(7) $${\left[\sum_{y\in N(x)}\left|d_{xy}-\bar{d_{x}}\right|\right]}^{2}\leq \left|N\left(x\right)\right|\sum_{y\in N(x)}{\left(d_{xy}-\bar{d_{x}}\right)}^{2}$$

As the result, 0 ≤ *scw_x_* ≤ 1. The definition of *scw_x_* takes into consideration the average spatial distance deviation between node *x* and *N*(*x*). Thus, the larger value of *scw_x_* is, the smaller difference variation between node *x* and *N*(*x*). That is, node *x* has higher spatial correlation with *N*(*x*) as its *scw_x_* is larger.

Based on the measurements, there are two possible situations when a node should be assigned as a determined/candidate one of virtual cluster head (*vch*):

1. A node has very low *scw*. (*lb* – *vch*, a determined *vch* satisfying the predefined lower bound *scw^lb^*)
2. A node has very high *scw.* (*ub* – *vch*, a candidate *vch* satisfying the predefined upper bound *scw^ub^*)

**Figure 3.**
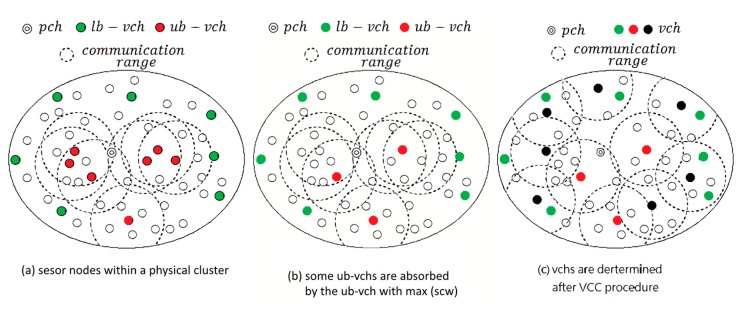
The role transition of nodes during virtual cluster construction (VCC) process. (**a**) sensor nodes within a physical cluster; (**b**) some *ub-vchs* are absorbed by the *ub-vch* with max (scw); (**c**) vchs are determined after VCC procedure.

Obviously, nodes being in either of two situations should be conditionally chosen as the representative node for a virtual cluster, depicted in Figure 3a. For the first situation, a node becomes *lb* – *vch* due to its low *scw*. This means that the data readings of the node are evidently different from its neighbors. Taking into consideration the application accuracy and integrity, a low *scw* node should be still awake in monitoring the environment. For the second situation, the upper bound of the weight is used to make sure that the nodes having higher correlation with their neighbors are chosen to be *ub* – *vch*s. However, it is possible that neighboring *ub* – *vch*s are highly correlated. They should be grouped into a virtual cluster, as depicted in Figure 3b. Noted that the $scw_{x}^{\text{max}}$ is calculated at *pch* side and possible *vch*s are selected in a centralized manner. As to virtual cluster construction (VCC) procedure, the virtual clusters are constructed in a distributed manner oppositely. If a node can join several clusters, it has to choose the nearest *vcw* to join. The results can refer to Figure 3c. The details of VCC procedure are described as follows.

After the virtual cluster construction, the representative nodes have to bear the monitoring responsibility. Meanwhile, the energy consumption for readings transmission is effectively reduced because of the representative mechanism. That is to say, the rest of nodes simultaneously turn their radio and sensing unit off and the energy are further conserved due to the contribution of the representative nodes.

Virtual cluster heads selection (VCHS) procedure:
Input: (*R_s_*(*t*_1_), *R_s_*(*t*_2_), …, *R_s_*(*t_k_*)) of sensor node *s* ∈ *S*, lower bound and upper bound of spatial correlated weight *scw_x_* for any node *x* ∈ *S*Output: the set *V*′, *V*″ of virtual cluster heads (*vch*) of *S*Step1: *V*′, *V*″ = ØStep2: calculation of spatial correlated weight *scw_x_* for any node *x* ∈ *S*Step3: on *lb* − *vch* (*V*′ = Ø)if (*scw_x_* ≤ *scw^lb^*) *V*′ = *V*′ + *x*;Step4: on *ub* − *vch*(*V*″ = Ø) for each node *x*,
$scw_{x}^{\text{max}}=\max\{scw_{y}|y\in N(x)\}$ if (*scw_x_* ≥ *scw^ub^* and
$scw_{x}>scw_{x}^{\text{max}}$) *V*″ = *V*″ + *x*;

Virtual cluster construction (VCC) procedure:
Step1: each node *x*(*x* ∈ *V*″) broadcasts an *indicator* message to *N*(*x*)Step2: each node *x* (*x* ∈ {*S*−V′−*V*″} receives no *indicator* message after a predefined time period, *x* broadcasts an indicator message to *N*(*x*)Step3: node *y*(*y* ∈ {*S*−V′−*V*″}) joins the virtual cluster of *x*(*VC_x_*): 3-1: if *y* receives only one *indicator* message from x 3-2: if *y* receives more than two *indicator* messages from *N*(*y*), then it joins *VC_x_* if *d_xy_* = min{*d_xy_*|*x* ∈ *N*(*y*)}Step4: if node *y* decides to join *VC_x_* it sends a *join* message to node *x*Step5: if node *x* receives a *join* message, it sends back an *acknowledge* message to *y*. Hence *x* is the virtual cluster head (*vch*) of *VC_x_* and *y* is a member of *VC_x_*.

## 4. Simulation Results

In this section, we develop a simulator in C++ for the evaluation of the proposed algorithm. To investigate the efficiency of energy conservation and the accuracy of monitoring environment, LEACH and 2TC-cor are compared in the experiments. HNA [29] denoted as the estimated value for the sensing intervals in which half of the nodes die is used as the metric to evaluate the efficiency of energy conservation. As to the accuracy of monitoring environment, some check points are predefined in order to examine the difference between the forecasting/representative readings and the concrete data readings. The difference is used as the metric of the accuracy of monitoring environment. Details of the simulation environment are given in Table 1. The nodes are randomly distributed in a square region and the sink node is located in the center of the region. Once the cluster members gather the data readings (sense the environment), they report to cluster head within the sensing interval. Particularly, the sensing interval is set to five seconds in order to speed up the observation of the simulation. Besides, the pre-specified basis function (*F*) is associated with k value to predict the sensing environment in our scenario. A scenario is simulated 30 times to produce more reliable results and the average of results is taken.

### 4.1. Efficiency of Energy Conservation

**Figure 4.**
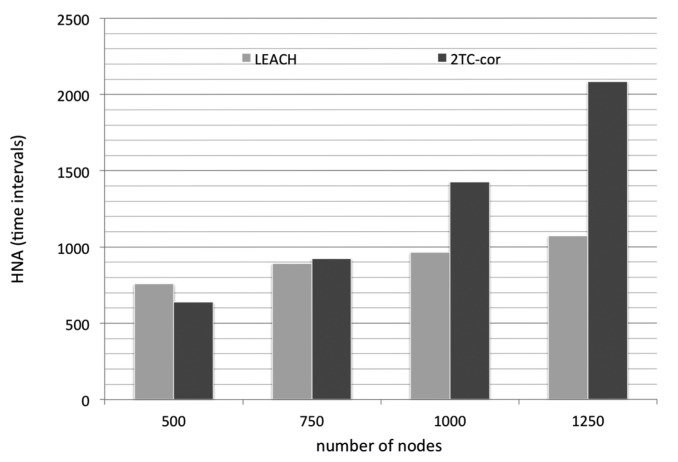
The time at which half of the node are dead.

We first evaluate the efficiency of energy conservation by comparing the HNA of the two algorithms (LEACH and 2TC-cor). Simply, the LEACH protocol is a hierarchical protocol in which nodes transmit their data readings to cluster heads. The mechanism of cluster head rotation leads to a balanced energy consumption and hence to a longer lifetime of the network. Obviously, the denser networks have the longer lifetime as shown in Figure 4. But the cluster members are continuously sensing the environment and reporting to cluster head. It leads to unnecessary energy consumption while the reported data are highly temporal and/or spatial correlated.

Our 2TC-cor algorithm uses prediction model to reduce the transmission in which the reporting data are highly temporal correlated. Also, some nodes can turn off their radio units (*i.e.*, avoid idle listening) for more energy saving based on the prediction mechanism. Furthermore, the nodes with highly spatial correlated data are grouped into virtual clusters. In the context, only the representative node is working and the neighboring nodes within a virtual cluster rest instead. It is rational that the representative mechanism results in the reduction of energy consumption, especially under a denser network. However, the overhead of constructing virtual clusters is non-negligible. In the experiments of sparser network, the expended energy for constructing virtual clusters exceeds the conserved energy by the predicting and representative mechanism. Therefore, the performance of our algorithm is not as good as LEACH in the metric of energy efficiency under the sparser network condition (number of nodes are 500 and 750). But, the benefits of energy conservation reveal finally under the denser network condition (number of nodes are 1000 and 1250) as depicted in Figure 4.

### 4.2. Accuracy of Monitoring Environment

Next, we focus on the accuracy of monitoring environment. Due to the prediction and representative mechanism, the possible difference between the forecasting/representative and concrete data readings comes from the reduced transmission. Therefore, in order to investigate 2TC-cor accuracy, we predefine 4 check points to examine the difference. The check points are set at each 82 time intervals. The aim is to make the check points set at the Δ period, seen in Figure 2.

In order to collect the concrete data readings, 2TC algorithm should be adaptively altered. It is worth noting that the representative mechanism should be ignored during the simulation of altered 2TC-cor. That is to say, not only the representative node (*vch*) works but all of the cluster members in a virtual cluster have to work (*i.e.*, sense the environment and report to *vch*). Besides, the prediction model should be ignored, too. Each *vch* report continuously its data readings to its *vch* instead of reporting only while “violations” appearing (reporting occasionally based on the prediction model).

The difference between reported readings from virtual cluster member nodes (*i.e.*, concrete data reading) and sensed readings of *vch* (*i.e.*, the representative node) is used to examine the 2TC-cor accuracy. We denote the average difference (*d̄*(*t*)) at *vch* side as

(8) $$\bar{d}\left(t\right)=\frac{\left|VC\right|-\sum_{i}^{VC}\frac{\left|R\left(t\right)-R_{i}\left(t\right)\right|}{R\left(t\right)}}{\left|VC\right|}$$

where *R*(*t*) is the sensed readings of *vch, R_i_*(*t*) is the concrete data reading from virtual cluster member node *i* and |*VC*| is the number of virtual cluster member nodes. Finally, the accuracy index of our algorithm is defined as:

(9) $$\text{accuracy index}=\sum_{x}^{N}\frac{\frac{\sum_{y}^{S}\bar{d}}{\left|S\right|}}{\left|N\right|}$$

where *x* and *y* are *pch* and *vch*, |*N*| and |*S*| are the number of physical and virtual cluster heads, respectively.

**Figure 5.**
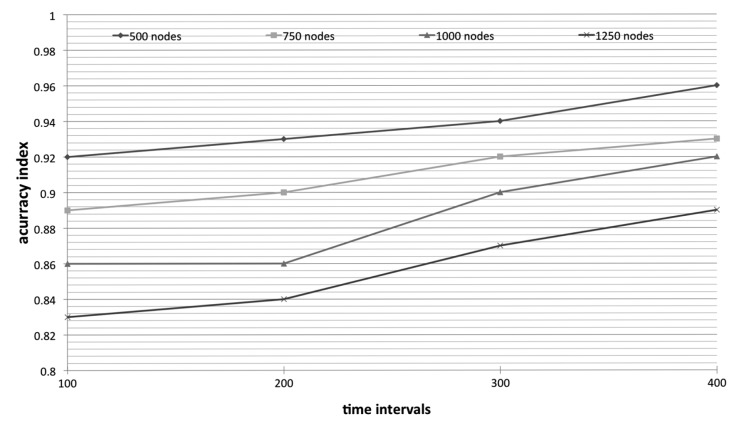
The accuracy of 2TC-corr in monitoring environment.

Therefore, the accuracy index of our algorithm is obviously lower than LEACH because of the prediction and representative mechanism. As shown in Figure 5, the accuracy index is progressively increasing as the times goes by (or say nodes are dying). Naturally, nodes are gradually draining their energy. In order to accurately monitor the whole environment, more nodes are expected to work while more nodes are dead. In other words, as the working nodes are getting fewer, the accuracy index is getting higher due to the efficiency of representative mechanism is not as active.

## 5. Conclusions

While saving energy, there are three key characteristics of WSNs that must be carefully maintained: quality of monitoring, reachability in the network, and communication delay. Due to the underlying hierarchical routing protocol, the reachability and communication delay can be disregarded in our algorithm rationally. Therefore, we just focus on the efficiency of energy conservation and accuracy of monitoring environment. In this article, we propose 2TC-cor with consideration of the spatial and temporal correlation of data readings. As presented by simulation results, it can effectively conserve energy by the prediction and representative mechanisms. However, the accuracy of monitoring environment must be carefully investigated. Besides, with consideration of various applications, the representative mechanism or virtual clusters formation should take the residual energy of nodes into account in our future work.

## Figures and Tables

**Table 1.** Simulation environment.

**Parameter**	**Value**
Network size	1000 m × 1000 m
Number of nodes	500, 750, 1000, and 1250
Signal range	150 m
Sensing interval (t^*^)	5 (s)
Ratio of Δ to *k*(*k* = 72)	1/6
Pre-specified basis function (*F*)	*F* = {1, *t, t*^2^}
Error threshold ε (|*R̂s*(*t*) – *Rs*(*t*))	0.1*R_s_(*t*)
Upper bound of spatial correlated weight (*scw^ub^*)	0.8
Lower bound of spatial correlated weight (*scw^lb^*)	0.2
